# Dissecting aneurysm of arch and descending thoracic aorta presenting as a left sided hemorrhagic pleural effusion

**DOI:** 10.4103/0970-2113.71963

**Published:** 2010

**Authors:** Shelley Shamim, Sumitra Basu Thakur, Amitava Sengupta, Sujit Kumar Bhattacharyya, Niranjan Sit

**Affiliations:** *Department of Chest Medicine, Nil Ratan Sirkar Medical College and Hospital, Kolkata, West Bengal, India*

**Keywords:** Dissecting aneurysm, hemorrhagic pleural effusion, CT angiography, diagnostic imaging

## Abstract

The most common cause of massive hemorrhagic effusion is malignancy. Herein we present a case of dissecting aneurysm of descending thoracic aorta presenting initially with shortness of breath due to left sided massive pleural effusion. Effusion was hemorrhagic in nature with high hematocrit value. CT scan of thorax with CT angiogram was done and that revealed the diagnosis.

## INTRODUCTION

Hemorrhagic pleural effusion is one of the common clinical problems of day to day practice. Apart from trauma, malignancy and, pulmonary embolism, are the leading causes of hemorrhagic pleural effusion.[1] Dissecting aortic aneurysm is rather an uncommon diagnosis in a patient with hemorrhagic effusion. Hemothorax is found hardly in 10% of aortic dissection and are mostly found in dissection of descending thoracic aorta.[2–4] In dissecting aneurysm, an intimal flap is produced making a false lumen in media and that may again rupture into the pleural space producing hemothorax.

## CASE REPORT

**Figure 1:**
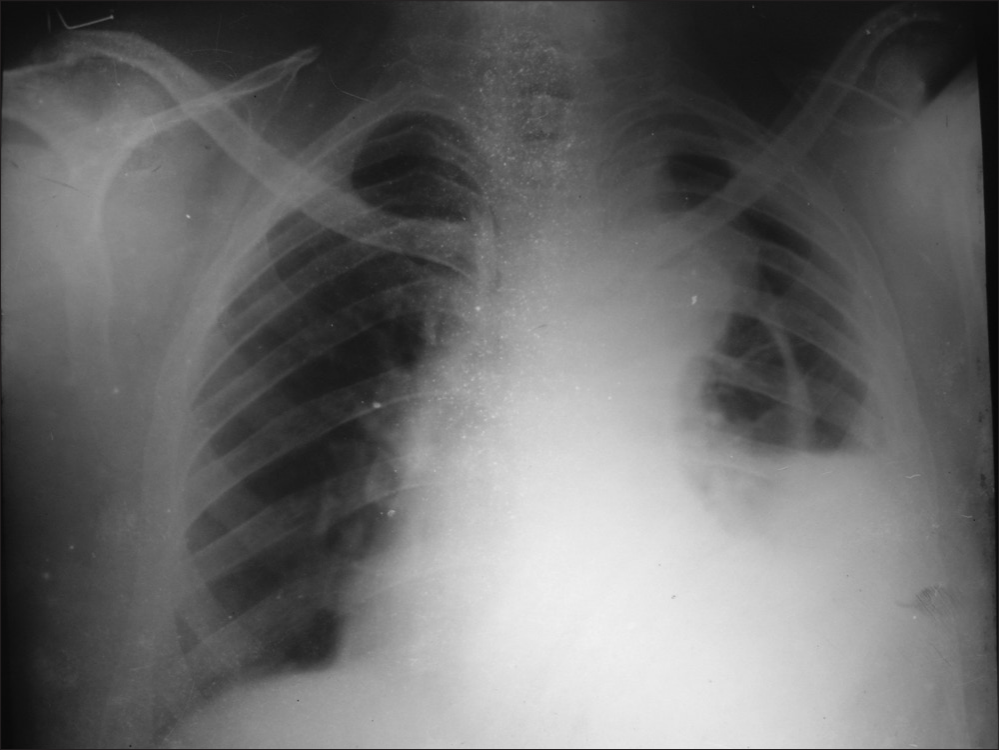
Left sided effusion with a dense homogenous opacity near aortic knuckle

**Figure 2:**
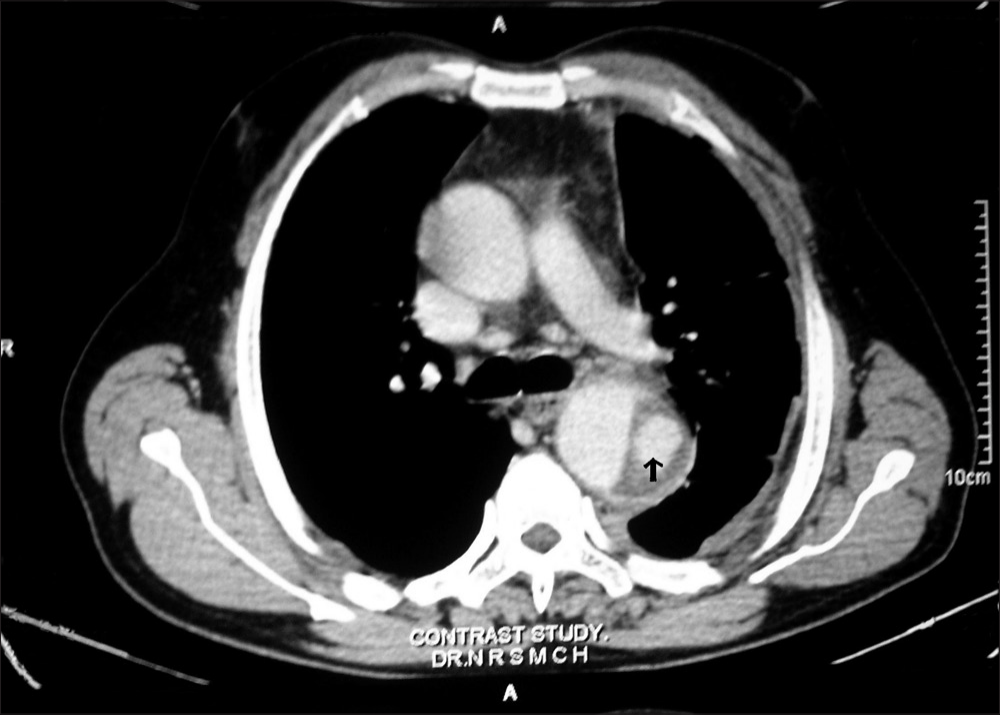
Dissecting aneurysm of descending aorta with false lumen (black arrow)

**Figure 3:**
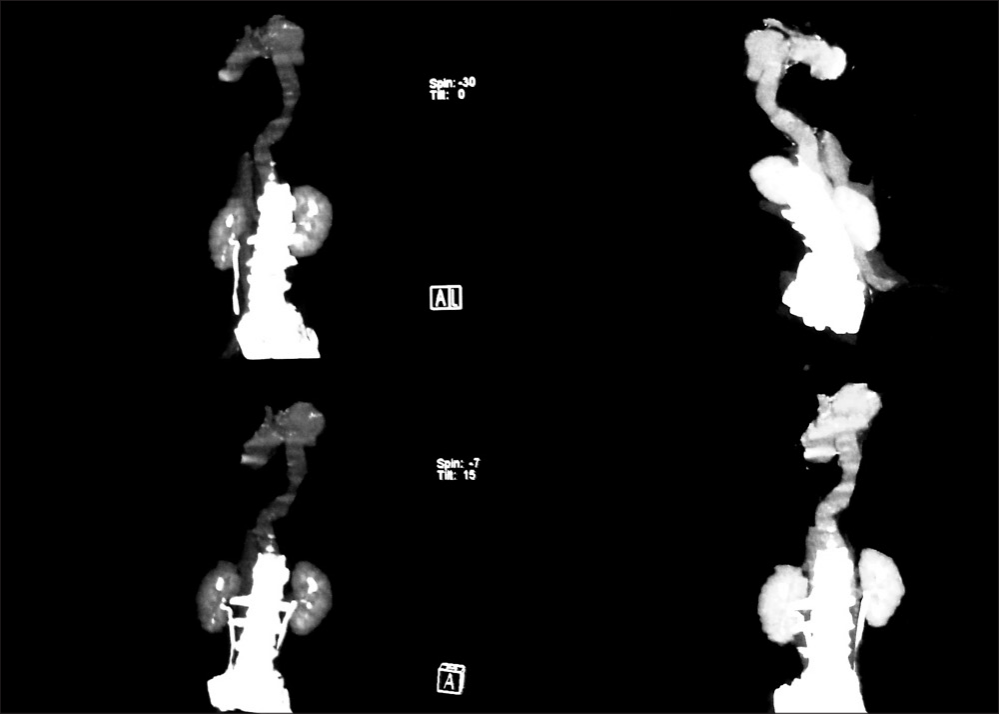
CT aortogram shows aneurysm of arch and descending aorta

A fifty-year-old, non diabetic, hypertensive, smoker male attended in our emergency with a MRC grade III dyspnea on 25.11.2008. His shortness of breath was acute in onset and started 12 days back. It was associated with chest pain which was left sided, sudden in onset, radiated to back and was burning in nature for the same duration. There was no significant history of cough, fever, palpitation, hemoptysis, pain in calf muscle, weight loss or any paroxysmal nocturnal dyspnoea. There was no history of bleeding disorder. He was hypertensive and took amlodipine 5 mg regularly for the last two years. He was a chronic smoker (used to take 20 pack bidi/yr). At the time of admission, on examination, the patient was tachypneic (respiratory rate 32/min), pulse rate was 100/min regular, equally palpable in all four limbs., blood pressure was 150/90 mm Hg in supine position at left arm and there was no difference with the right arm reading. The lower limb pressure was equal with upper limb. There was no pallor found. Upper respiratory tract examination was within normal limit. There was decreased movement in left hemi thorax. Trachea was shifted to the right, apex beat couldnot be localized. Vocal fremitus on left was decreased, dull percussion note was noted on the same side in whole hemithorax. Traube’s space percussion was dull. On auscultation breath sound and vocal fremitus were diminished on left side. Examination of other systems didnot reveal any abnormality. On routine investigation, his hemoglobin was 13.4 gm%, TLC 7800/cc. N70% L49% M6% E5%, platelets adequate, ESR 36 mm/1^st^h. Urea 18 mg/dl, creatinine 0.9 gm/dl, sugar 91 mg/dl. X-ray dated on 23.11.08 revealed massive pleural effusion on left side with shifting of mediastinum to right. ECG showed sinus tachycardia only. Pleural fluid aspiration over 800 ml was done and was haemorhagic in nature, cytology 705/cc. cell type L60%N35%E5%, RBC was fair in number, protein 3.95 mg/dl, ADA 36iu, ZNstain No AFB found, Gram stain no bacteria, PAP smear shows no Malignant cell. Pleural fluid haematocrit was 27% while serum haematocrit was 41%. Repeat x-ray after pleural aspiration shows left sided effusion with a dense homogenous opacity near aortic knuckle [Figure 1]. Sputum for AFB for three consecutive days was negative. CT scan of thorax and CT angiography with 3 D reconstructions [Figure 2 and 3] shows dissecting aneurysm of arch and descending thoracic aorta with leaking post wall. Pleural fluid was aspirated with repeated aspirations and patient improved with medical management; he was put on atenolol 100 mg OD and ACE inhibitor and is currently doing well in 8 months of follow up.

## DISCUSSION

Rupture of dissection of aorta has a very high mortality of > 50%.[2,5] There are only few reported cases where a chronic and survived case of ruptured dissection of aorta presented initially with only massive hemorrhagic pleural effusion,[6] so we report this case. Aortic dissection is caused by a circumferential or, less frequently, transverse tear of the intima. There are at least two important variants of aortic dissection: intramural hematoma without an intimal flap and penetrating atherosclerotic ulcer with an intimal flap. The important factors that predispose to aortic dissection are systemic hypertension (a coexisting condition in 70% of patients), cystic medial necrosis, Marfan syndrome, congenital aortic valve anomalies (e.g., bicuspid valve) and aortic trauma.[7] Our patient had preexisting hypertension. Aortic dissection is classified with Stanford classification where it is either type A, in which the dissection involves the ascending aorta (proximal dissection), or type B, in which it is limited to the descending aorta (distal dissection). From a management standpoint, classification of aortic dissections into type A or B is more practical and useful as type A dissections require surgery, while type B dissections may be managed medically under most conditions.[7] Our case had type B dissection and we could manage him medically. Acute aortic dissection presents usually with sudden onset of pain, which is often described as very severe and tearing and is associated with diaphoresis, but pain was not severe in our case and it was not even the main presenting complaint. He was mainly presented with shortness of breath with left sided massive hemorrhagic pleural effusion and even the X-ray done after pleural tap revealed a mass-like opacity near aortic knuckle, so malignancy was the main differential diagnosis. However, the onset was acute in nature and opacity near aortic knuckle may be an aneurysm; therefore, dissecting aneurysm was considered and CT scan of thorax revealed the diagnosis. Most of the cases that are reported where leaking dissecting aneurysm initially presented with massive hemorrhagic effusion had anemia and low hematocrit value,[6,8,9] but hemoglobin was 13.5 gm% in our case and patient was hemodynamically stable and that is the peculiarity of our case.
